# The importance of supplementary immunisation activities to prevent measles outbreaks during the COVID-19 pandemic in Kenya

**DOI:** 10.1186/s12916-021-01906-9

**Published:** 2021-02-03

**Authors:** C. N. Mburu, J. Ojal, R. Chebet, D. Akech, B. Karia, J. Tuju, A. Sigilai, K. Abbas, M. Jit, S. Funk, G. Smits, P. G. M. van Gageldonk, F. R. M. van der Klis, C. Tabu, D. J. Nokes, LSHTM CMMID COVID-19 Working Group, James D. Munday, Carl A. B. Pearson, Simon R. Procter, Oliver Brady, David Simons, Rachel Lowe, W. John Edmunds, Katharine Sherratt, Rosanna C. Barnard, Alicia Rosello, Adam J. Kucharski, Fiona Yueqian Sun, Nikos I. Bosse, Petra Klepac, Yang Liu, Kiesha Prem, Gwenan M. Knight, Akira Endo, Sam Abbott, Emily S. Nightingale, Thibaut Jombart, Jon C. Emery, Georgia R. Gore-Langton, Joel Hellewell, James W. Rudge, Hamish P. Gibbs, Kathleen O’Reilly, Kevin van Zandvoort, Yung-Wai Desmond Chan, Damien C. Tully, Anna M. Foss, Christopher I. Jarvis, Katherine E. Atkins, Samuel Clifford, Matthew Quaife, Billy J. Quilty, Rein M. G. J. Houben, Rosalind M. Eggo, Graham Medley, Sophie R. Meakin, Timothy W. Russell, Nicholas G. Davies, Charlie Diamond, Arminder K. Deol, C. Julian Villabona-Arenas, Stéphane Hué, Megan Auzenbergs, Quentin J. Leclerc, Amy Gimma, JAG Scott, S. Flasche, IMO Adetifa

**Affiliations:** 1 KEMRI-Wellcome Trust Research Programme, Kilifi, Kenya; 2 Department of Infectious Diseases Epidemiology, London School of Hygiene and Tropical Medicine, London, UK; 3 Department of Immunosurveillance, Centre for Infectious Diseases Control, National Institute of Public Health and the Environment (RIVM), Bilthoven, The Netherlands; 4 National Vaccine and Immunisation Programme, Ministry of Health, Nairobi, Kenya; 5 School of Life Sciences and Zeeman Institute for Systems Biology and Infectious Disease Epidemiology Research (SBIDER), University of Warwick, Coventry, UK

**Keywords:** Measles, Vaccination coverage, Outbreak, COVID-19, Supplementary immunisation activities

## Abstract

**Background:**

The COVID-19 pandemic has disrupted routine measles immunisation and supplementary immunisation activities (SIAs) in most countries including Kenya. We assessed the risk of measles outbreaks during the pandemic in Kenya as a case study for the African Region.

**Methods:**

Combining measles serological data, local contact patterns, and vaccination coverage into a cohort model, we predicted the age-adjusted population immunity in Kenya and estimated the probability of outbreaks when contact-reducing COVID-19 interventions are lifted. We considered various scenarios for reduced measles vaccination coverage from April 2020.

**Results:**

In February 2020, when a scheduled SIA was postponed, population immunity was close to the herd immunity threshold and the probability of a large outbreak was 34% (8–54). As the COVID-19 contact restrictions are nearly fully eased, from December 2020, the probability of a large measles outbreak will increase to 38% (19–54), 46% (30–59), and 54% (43–64) assuming a 15%, 50%, and 100% reduction in measles vaccination coverage. By December 2021, this risk increases further to 43% (25–56), 54% (43–63), and 67% (59–72) for the same coverage scenarios respectively. However, the increased risk of a measles outbreak following the lifting of all restrictions can be overcome by conducting a SIA with ≥ 95% coverage in under-fives.

**Conclusion:**

While contact restrictions sufficient for SAR-CoV-2 control temporarily reduce measles transmissibility and the risk of an outbreak from a measles immunity gap, this risk rises rapidly once these restrictions are lifted. Implementing delayed SIAs will be critical for prevention of measles outbreaks given the roll-back of contact restrictions in Kenya.

**Supplementary Information:**

The online version contains supplementary material available at 10.1186/s12916-021-01906-9.

## Background

The SARS-CoV-2 pandemic has damaged the economy and disrupted social interaction and important health services in Kenya and elsewhere [1, 2]. The cumulative incidence of COVID-19 cases continues to rise in many parts of Africa suggesting the current mitigation measures will be maintained or reintroduced for periods at least until the pandemic peaks [3].

Despite the World Health Organization (WHO) advisory to sustain routine immunisation (RI), vaccine coverage temporarily declined in many countries including Kenya that reports a 33% disruption of RI [4–7]. Following guidance from the WHO, all countries suspended scheduled measles SIAs [6–8]. Measles control in Kenya is achieved by giving children a first dose of measles-containing vaccine (MCV1) at 9 months, and a second dose (MCV2) from 18 months. SIAs, first introduced in 2002, are conducted periodically among children < 5 years or < 15 years for accelerated control of measles [9]. Based on the accumulation of susceptible children, the timing of such campaigns has typically been chosen to close immunity gaps in time to prevent potentially large measles outbreaks. A measles SIA originally planned for 2019 was rescheduled for February 2020 due to a shortfall in funding and postponed again following the COVID-19 pandemic.

Following identification of the first COVID-19 case on March 13, 2020, Kenya imposed various mitigation measures: ban on large gatherings, suspension of international flights, closure of bars, cessation of movement from hotspot counties, restriction of restaurant operating hours, and a nationwide curfew from 7 pm to 5 am. While it is plausible that these physical distancing and lock down measures may reduce the risk of measles outbreaks, they are temporary and may be associated with rebound risk periods.

The availability of recent measles serological data provided the opportunity to use Kenya as a case study to estimate the impact of reduced measles vaccination coverage and suspended SIAs due to COVID-19 on the risk of measles outbreaks.

## Methods

This study used a cohort mathematical model that combined measles serological data, local contact patterns, and vaccination coverage estimates.

### Serological data

We estimated measles immunity profile in children using serum samples collected during serological surveys among residents of Kilifi Health and Demographic Surveillance System (KHDSS) Kilifi, Kenya [10] for the Pneumococcal Conjugate Vaccine Impact Study (PCVIS) [11]. These serosurveys, conducted every 2 years since 2009, target 50 KHDSS randomly selected children in ten age strata (0, 1, 2, 3, 4, 5, 6, 7, 8–9, and 10–14 years) and blood samples < 2 ml were collected from participants. The sample size for the PCVIS serosurveys was calculated to obtain narrow confidence intervals around the estimate of prevalence of immune response both overall and by age-category for each serosurvey year. For instance, for a proportion of 0.80, the 95% confidence intervals (CIs) would be 0.77–0.84 overall and 0.69–0.91 in each age stratum.

In the 2019 serosurvey, there were 497 participants and the blood samples were collected in July (165), August (162), September (130), and October (40). We tested for measles immunoglobulin G (IgG) antibodies using a fluorescent-bead-based multiplex immunoassay. Antibody concentrations ≥ 0.12 IU/ml were considered protective against measles [12].

We assumed these results reflected measles immunity in Kilifi in August 2019 and assumed 96% of persons > 15 years had protective measles antibodies concentrations, similar to findings in adults in Nairobi in 2007–2009 [13] (Table 1). We also assumed protection from maternal immunity was similar to the proportions of the infants < 9 months old who had protective antibodies.

**Table 1:** Model parameters. An overview of the key model parameter assumptions and their sources. Parameter ranges are those used in the sensitivity analyses

Parameter	Value (95% quantiles)	Source
Vaccine schedule	MCV1: 9 months MCV2: 18 months	[14]
Vaccine effectiveness (beta distributed)	MCV1: 85% (80–90%) +MCV2: 98% (95–100%) Combined effectiveness 93% (88–96%)	[15, 16]
Age-immunity profile in < 15 years old (bootstrapped from data)	Observed in 2019	[11]
Proportion immune among > 15 years old (beta distributed)	96% (90–99%)	[13]
Vaccine coverage August 2019 to March 2020 (assumed to be same as in 2018) (beta distributed)	MCV1: 79% (75–85%) MCV2: 45% (40–50%)	[9, 17, 18]
Vaccine coverage from April 2020	MCV1 and MCV2 0%, 15%, 50%, or 100% reduced	Assumption
*R*_0_ measles (Log-normally distributed)	14 (12–18)	[19]
Reduction in contacts during COVID-19	50% (25% and 75%)	[20]
Age demographics	From KHDSS in 2019	[10]
Social mixing matrix	From 2011/12	[21]

### Vaccination coverage

MCV1 national coverage in Kenya has been between 75 and 80% since its introduction in 1985 [17]. MCV2 was introduced in Kenya in 2013 and coverage rose up to 45% in 2018 [9]. The last measles SIA in children aged 9 months to 14 years took place in 2016 and achieved 95% coverage [22].

We assumed national MCV1 and MCV2 coverage were 79% and 45%, respectively, in 2018, and that these stayed at the same level from August 2019 until the end of March 2020 when COVID-19 contact restrictions were introduced in Kenya. From April 2020, we explored the following routine vaccination coverage scenarios alongside a suspended SIA.

A. Routine vaccination coverage remained the same
B. Routine vaccination coverage reduced by 15% for both MCV1 and MCV2
C. Routine vaccination coverage reduced by 50% for both MCV1 and MCV2
D. Routine vaccination was suspended

### Contact matrix

We used an age-mixing matrix which consisted of the number of contacts between six different age groups. The matrix was generated from diary studies conducted in Kilifi, Kenya [21], using a bootstrap of 4000 samples by randomly sampling n individuals with replacement from the n participants of the contact survey.

### Projecting immunity

We adapted a static cohort model of measles immunity [23] to estimate age-stratified population immunity profile in Kilifi by combining recent measles serological data with new vaccine-derived immunity during the prediction period using the local vaccination schedule, MCV1 and MCV2 uptake, and vaccine efficacy. We assumed waning immunity or additional acquired immunity from natural exposure, and demographic changes in the short time frame were negligible. Hence, the key mechanisms of the projection model were that individuals are born at a constant rate, gained immunity through vaccination at the recommended age and at the observed coverage, and grow older.

In extrapolating immunity for young infants under 9 months old, maternal immunity was assumed to be the same as the observed data. For ages 9 months to 17 months, immunity was estimated in accordance with the assumed MCV1 vaccination uptake and a vaccine effectiveness of 93%. For those ≥ 18 months, we estimated the immunity based on the assumed uptake of MCV2 and the same vaccine effectiveness. We aggregated projected immunity to age groups given by contact data and weighted each age group according to population estimates before averaging them to estimate overall immunity. We did not explicitly model MCV2 delivery but rather assumed that the MCV1 effectiveness is an average of MCV1 and MCV2 efficacy weighted by proportion of children who receive MCV1 only or both doses. The underlying assumption here was that the same children who received MCV2 had also received MCV1. We predicted age-stratified and population-level immunity until December 2021.

To derive a contact-adjusted estimate for the proportion of the population who are immune to measles, the predicted age-stratified immunity profile was weighted by age-stratified social contact patterns observed in Kilifi. This method has been previously shown to yield robust projections for measles immunity to transmission in the population [23].

The herd immunity threshold (HIT) for measles during the COVID-19 pandemic was calculated assuming an R_0_ of 12 to 18 with a median of 14 [19] and that COVID-19 prescribed contact restrictions caused a 50% reduction in measles transmissibility similar to the observed reduction in physical contacts in Kenya [20]. We also explored a 25% and 75% reduction in measles transmissibility in a sensitivity analysis. The HIT is calculated as 퓗_0_ = (*R*_0_–1)/*R*_0_.

### Quantitative impact of outbreak risk

We obtained a crude estimate of the outbreak risk using the predicted immunity and HIT. The probability of a large outbreak, *p*, sparked by a single infected individual was given by *p* = 1 − (1/*R*)^I0^ where I0 is the initial number infected and *R* is the effective reproductive number. *R* < 1 implies that probability, *p*, is negative which is defined to be 0 for no outbreak.

### The effectiveness of a post-lockdown SIA in reducing outbreak risk

We assessed the impact of SIAs in two age categories: 9 months to 5 years and 9 months to 15 years, by predicting the post-SIA immunity profile and the corresponding risk for a large measles outbreak. We simulated SIAs in either November 2020, December 2020 or December 2021, assumed a coverage of 95% similar to the most recent national SIA in 2016 [22], and applied vaccine efficacy of MCV1. The SIA was simulated by reducing the age-specific pool of susceptible by the effective coverage of the SIA.

In simulating the SIA, we used the age-specific predicted immunity to calculate the age-specific pool of susceptible at the different time-points. We reduced this age-specific pool of susceptible in the age-groups of interest by the effective coverage of the SIA. We aggregated the results and adjusted the overall crude immunity using the social contact matrix. Finally, we calculated the outbreak probability assuming a SIA is conducted before restrictions are lifted (using the reduced HIT based on 50% reduction in contacts) and assuming an SIA is conducted after restrictions are lifted (using the normal HIT based on 0% reduction in contacts).

### Uncertainty analyses

We assessed the sensitivity of our findings to uncertainty inherent in several of our assumptions via probabilistic re-sampling. We included uncertainty for population immunity profile, combined MCV1 and MCV2 vaccine effectiveness, and MCV1 and MCV2 coverage (Table 1). As part of each parameter bootstrap, we also bootstrapped participants of the serological survey and hence the age-stratified population immunity at the start of the simulation. We present median estimates including uncertainty quantified as per the 95% quantiles of the 4000 bootstrap samples.

### Sensitivity analyses

We conducted a sensitivity analysis to assess the impact of a delay in receipt of MCV1 on outbreak probability. We delayed the age of receipt of MCV1 in our model by 3 months as reported for delayed vaccination in Kilifi [18] and also by 6 months. We also predicted unadjusted population immunity in Kilifi and estimated the corresponding probability of a large outbreak.

## Results

### Measles seroprevalence in Kilifi in late 2019

**Fig. 1:**
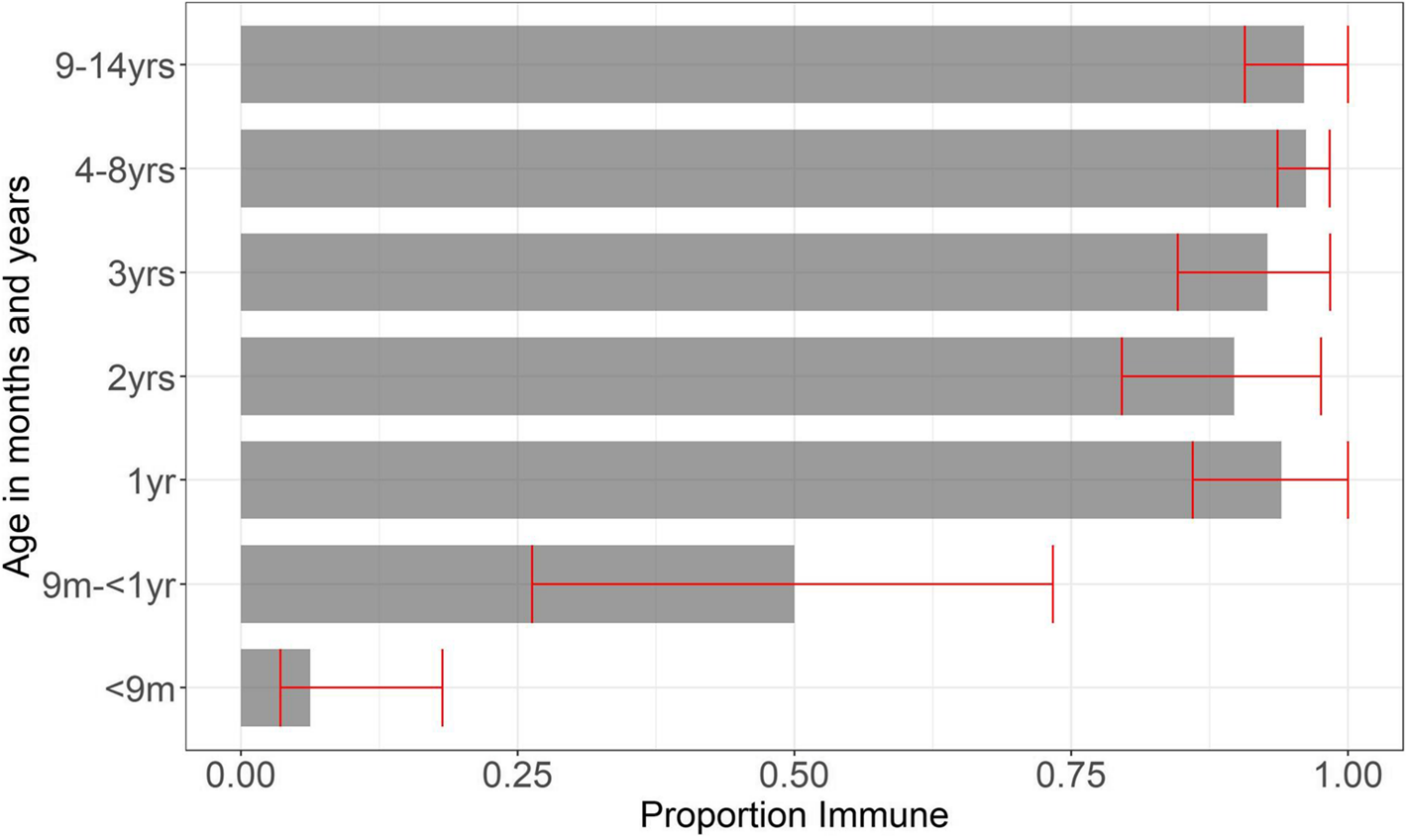
Age-stratified population immunity profile. Estimated age-stratified proportion of the Kilifi County population who were immune to measles infection in August 2019 from data. Antibody concentrations ≥ 0.12 IU/ml were defined as protective. Confidence bounds displayed (in red) are the 95% quantiles of a nonparametric bootstrap that is used to propagate uncertainty into the modelling framework. MCV1 is recommended to be administered at 9 months as per the Kenyan immunisation schedule and MCV2 from 18 months

The proportion of MCV1-eligible children with protective measles antibody concentrations was high in 2019 as shown in Fig. 1 and Additional file 1; Table S1. Seventy-one of 74 (96%) children ≥ 9 years had protective levels. Similarly, 228 of 237 (96%) 4–8-year-olds were immune. Among under-fours eligible for MCV1, 145 of 166 (87%) were immune while one of 20 (0.05%) children under 9 months old, who were ineligible for MCV1, had protective antibodies.

### Age-adjusted immunity

We estimate that in late 2019, population immunity adjusted for age-differences in social contacts was 90% (85–92). Predicted immune proportions were unchanged in February 2020, at the time of originally planned SIA.

**Fig. 2:**
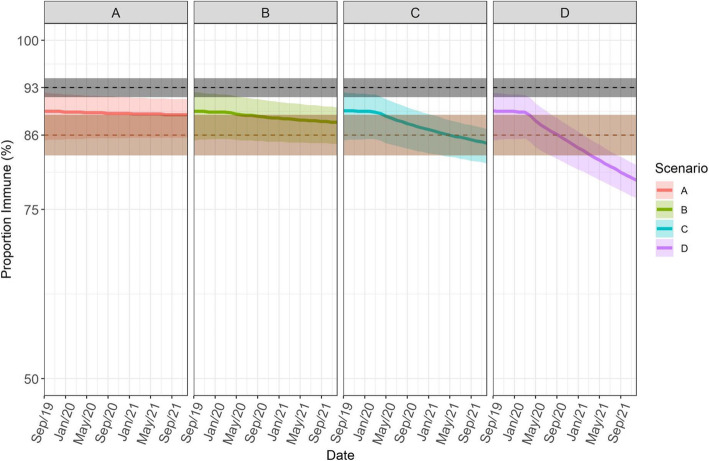
Monthly projected age-adjusted immunity profiles from September 2019 to December 2021. The changes in coverage took effect in April 2020. The black dotted line shows the herd immunity threshold for measles before the COVID-19 physical distancing measures, 0.93[0.92-0.94] and the brown dotted line shows the herd immunity threshold during COVID-19 physical distancing measures, 0.86[0.83–0.89], assuming the lockdown measures are still in effect. The bold lines and shaded region in each scenario, i.e. **a**. no reduction, **b**. 15% reduction, **c**. 50% reduction, and **d**. 100% reduction, indicate the median estimates and the uncertainty of the predicted immunity quantified as the 95% quantiles of the bootstrap analysis. There was a quick decline of predicted immunity over the study period that was based on assumed reduction in routine coverage

Following the start of COVID-19 pandemic and restriction measures that caused a decrease in vaccination coverage, we estimate that population immunity decreased quickly, depending on the extent of reduction in vaccination coverage. If coverage reduced by 15% from April 2020, the contact-adjusted population immunity would decline to 88% (85–91) by December 2020 and 87% (84–90) by December 2021. A 50% reduction in vaccination coverage would lead to a more rapid decline in this immunity to 87% (83–89) in December 2020 and 85% (81–87) in December 2021(Fig. 2).

### Age-adjusted immunity vs herd immunity threshold

A basic reproduction number of 14 (12–18) implies a HIT of 93% (82–94) and if, as a result of physical distancing, measles transmission is reduced by 25%, 50%, and 75% this HIT drops to 90% (89–93), 86% (83–89), and 71% (67–78) as seen in Additional file 2: Fig. S1. Before contact restrictions came into effect in April 2020, age-adjusted immunity was below the HIT: in 99% of simulations this immunity was below the HIT (Additional file 3: Fig. S1). Reduction in HIT temporarily mitigated the immediate risk for measles outbreak as in April 2020, 94% of simulations were above the 50% reduced transmission HIT, 20% were above the 25% reduced HIT, and 100% of simulations were above the 75% reduced HIT.

Depending on vaccination coverage maintained during COVID-19 pandemic, population immunity may decline quickly in young children (< 2 years). By April 2020, age-adjusted immunity fell below the normal transmission HIT in all simulations under all the scenarios. (Additional file 3: Fig. S1).

**Fig. 3:**
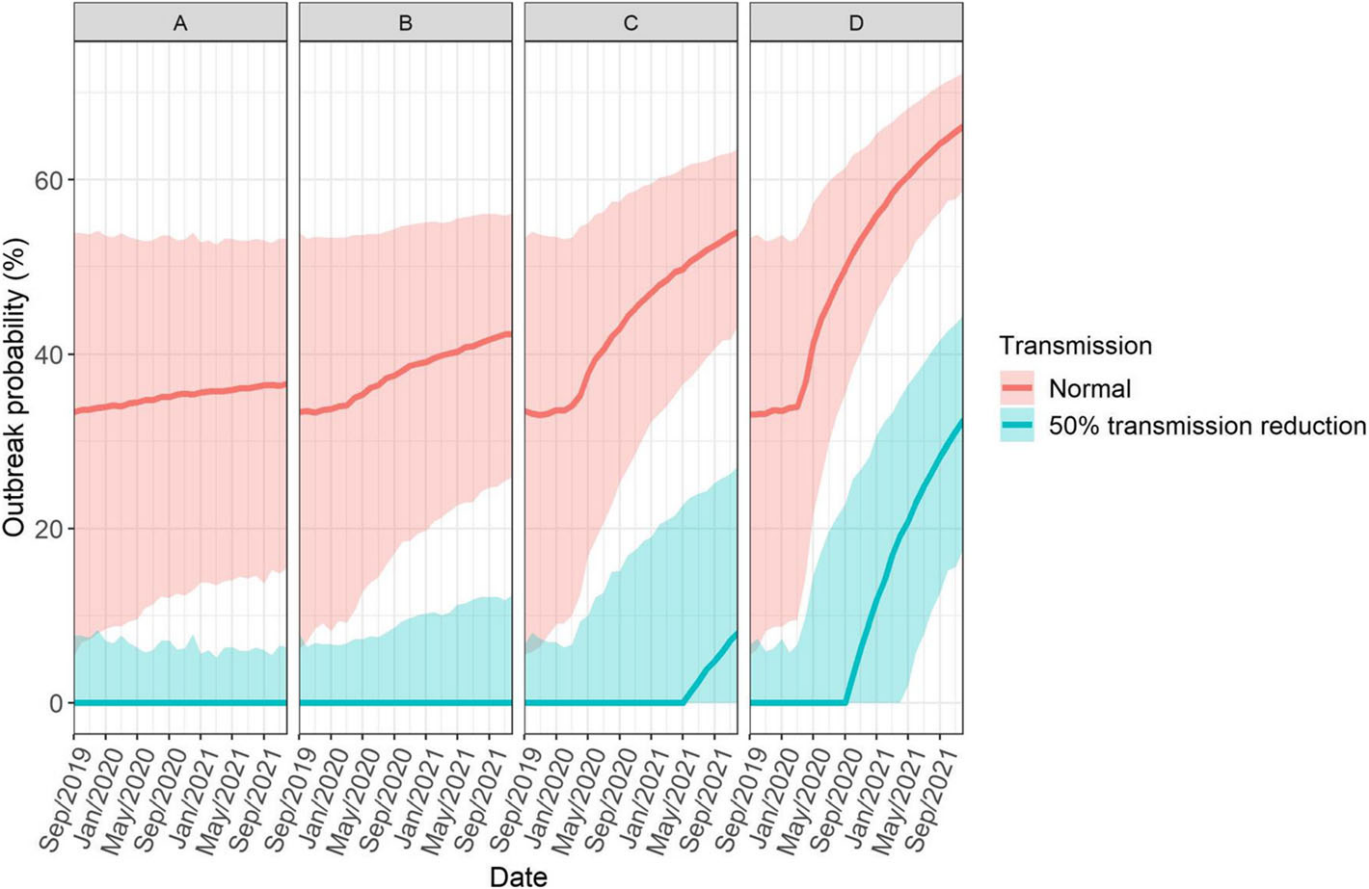
Probability of a large measles outbreak sparked by a single infected individual. Outbreak probability was calculated using the predicted immunity and herd immunity threshold before (red) and during (green) COVID-19 movement restriction measures. Zero probability indicates no possibility of an outbreak. The bold lines and shaded region in each scenario, i.e. **a**. no reduction, **b**. 15% reduction, **c**. 50% reduction, and **d**. 100% reduction, indicate the median estimates of outbreak risk and the uncertainty quantified as the 95% quantiles of the bootstrap analysis. The risk of a large measles outbreak from the introduction of a single infectious individual increased quickly based on the level of impairment of routine vaccination coverage

Similarly, the risk of a large measles outbreak from the introduction of a single infectious individual increased quickly if routine vaccination coverage declined (Fig. 3). If in December 2020, measles transmissibility is similar to pre-COVID-19 levels and routine measles coverage since April 2020 reduced by 15%, 50%, or 100%, we estimate the probability for a large measles outbreak as 38% (19–54), 46% (30–59), and 54% (43–64), respectively, in the age-adjusted analysis. By December 2021, this risk would increase to 43% (25–56), 54% (43–63), and 66% (59–72), respectively. The probability of a large measles outbreak was much lower if measles transmissibility reduced by 25%, 50% and 75% (Additional file 4: Fig. S1). In December 2020, if routine measles coverage since April 2020 reduced by 50%, we estimate the probability of a large measles outbreak as 28% (7–45), 0% (0–18), and 0%(0–0) assuming a 25%, 50%, and 75% reduction in transmission.

### Effectiveness of a SIA

**Fig. 4:**
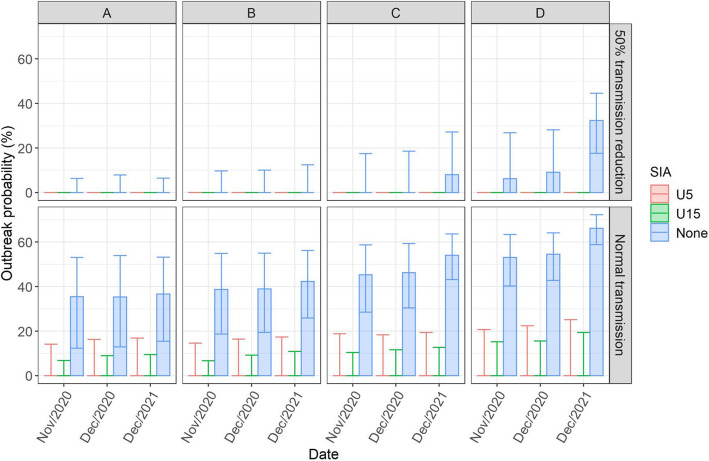
Probability of a single infectious person seeding a large outbreak before (none) and after implementing a SIA in children 9 months to 5 years old (U5) and in 9 months to 15 years old (U15) at different timepoints post-lockdown (normal transmission) and during lockdown (50% transmission reduction). Outbreak probability was calculated by comparing the proportion immune with the herd immunity threshold. The shaded area is the median estimate of the outbreak risk and the error bars indicate the uncertainty in outbreak risk quantified as the 95% quantiles of the bootstrap analysis. In all the scenarios, i.e. **a**. No reduction, **b**. 15% reduction, **c**. 50% reduction, and **d**. 100% reduction, the risk of a large measles outbreak would be largely mitigated through delivery of a SIA among children < 5 years old or < 15 years old

A SIA in 9-month to 5-year-old children or 9-months to 15-year-olds both during and immediately after lifting transmission-reducing COVID-19 restrictions can substantially reduce outbreak risk (Fig. 4).

If measles vaccine coverage declines by 15%, 50%, or 100% from April 2020, a post lockdown SIA delivered to children 9 months to 5 years old in December 2020 with 95% coverage would reduce the risk of an outbreak to 0% (0–17), 0% (0–20), and 0% (0–22), respectively, in age-adjusted analysis. A similar SIA would reduce the risk of an outbreak to 0% in all the scenarios assuming a 50% reduction in contacts in December 2020 (Fig. 4).

Even if RI coverage is low through to December 2021, the risk for a large measles outbreak would be mitigated through an SIA for under-fives if delivered as soon as possible (Additional file 5: Fig. S1).

### Impact of delayed vaccination on outbreak probability

A 3-month and 6-month delay in the receipt of MCV1 in age-eligible children caused a marginal increase in the risk of a large measles outbreak (Additional file 6: Fig. S1). This increase in outbreak risk associated with a delay in receipt of MCV1 was also evident for different assumptions of transmission reduction during lockdown (Additional file 6: Fig. S1).

### Crude population immunity

The predicted crude population immunity was slightly higher compared to age-adjusted immunity but followed the same declining trend over time (Additional file 7: Fig. S1). Before contact restrictions came into place, 73% of simulations were below the HIT and by October 2020 and July 2020, this immunity fell below the HIT in more than 95% of simulations under scenario C and D respectively (Additional file 8: Fig. S1).

## Discussion

Our analysis suggests a decline in population immunity during COVID-19 pandemic will result in an increased risk of a measles outbreak depending on the extent to which routine vaccination coverage is reduced. We estimated the probability of a large measles outbreak from the introduction of a single infectious individual to be 38% (19–54), 46% (30–59), and 54% (43–64) in December 2020 assuming a 15%, 50%, or 100% reduction in routine measles vaccination coverage respectively since April 2020. This risk, which will increase to 43% (25–56), 54% (43–63), and 67% (59–72) by December 2021, will be greatly reduced if a SIA among children < 5 years old is conducted before or immediately after all COVID-19-related restrictions on physical contact are lifted.

We based our analysis on an immunity model that combined serological data and age-specific mixing patterns in Kenya. Combining the two is a better strategy for predicting outbreaks as opposed to using immunity profiles alone as it allows adjustment of overall immunity by taking into account the contribution of each age-group to transmission [23].

As there is considerable uncertainty in actual reduction of routine vaccination uptake, we predicted population immunity for scenarios of routine vaccination coverage since April 2020, i.e. 15%, 50%, and 100% reductions, and the corresponding outbreak risk. Our assumption of 15% reduction in vaccine coverage rates is based on reduction in vaccine clinic visits in Kilifi County (DHIS2 Routine Report) while the 50% reduction lies in the range of reported disruption in vaccination services from WHO immunisation pulse poll [6]. We assumed a 50% reduction in measles transmissibility given that COVID-19 mitigation measures implemented on 25th March 2020 were reported to have reduced social contacts and disease transmission by the same margin [20]. Although some restriction measures remain in place, e.g. nationwide curfew, others like the partial lockdown have since been eased and ban on international flights was lifted on 1st August 2020. While the assumption of a 50% reduction in measles transmission was applicable at the beginning of the epidemic due to stringent measures imposed, current herd immunity threshold may be much higher than originally assumed but still lower than pre-COVID-19 threshold.

To account for the uncertainty in measles transmissibility during lockdown, we explored two other scenarios, 25% and 75% reduction in measles transmission in a sensitivity analysis. We found that a 75% reduction in measles transmission would result to zero outbreaks in all the scenarios during the entire study period, which was much lower compared to the outbreak probability in our baseline analysis. A 25% reduction in measles transmission resulted to a much higher probability of measles outbreak compared to our baseline analysis. For instance, in December 2020, the estimated outbreak risk was 28% (7–45) compared to 0% (0–18) in our baseline analysis assuming a 50% reduction in routine vaccination coverage.

In the calculation of a quantitative impact of outbreak risk, our estimate of the probability of a large outbreak was based on the introduction of a single infectious individual in a population where there is hardly any measles circulation. Based on our results, the outbreak probability would be much higher and severe if multiple cases were introduced.

SIAs in Kenya are generally conducted every 2–4 years and provide a second opportunity for vaccination in children regardless of their vaccination history and are ideally timed to close immunity gaps arising from the accumulation of susceptible and vaccine failures [24]. They have been shown to be effective in increasing immunisation equity by reaching children from poor households [25]. In February 2020, at the time of the planned national SIA, we estimated that 90% (85–92) of the population were immune after adjusting for age-differences in social contact. This immunity which was equivalent to a 34% (8–54) probability of a large outbreak suggests the SIA would have been timely in closing immunity gaps. The risk of an outbreak which was accelerated by immunity gaps arising in children who missed their routinely delivered MCV1 and MCV2 continued to increase in subsequent months following the start of COVID-19 and by December 2020, the estimated risk had increased to 38% (19–54), 46% (30–59), and 54% (43–64) assuming a 15%, 50%, and 100% reduction in measles vaccination coverage respectively. Based on limited information on additional reductions in vaccination coverage as the pandemic progressed in Kenya’s devolved counties and marked reduction in vaccination services in Kenya in May 2020 compared to January and February 2020 reported in the second WHO immunisation poll, it is highly probable most areas will experience an outbreak risk of 46% (30–59) corresponding to a 50% reduction in routine coverage.

Assuming all COVID-19 restrictions remain in place, the risk of outbreaks would only be experienced in the suspended RI scenario in 2021. The severity and timing of these outbreaks would be largely reduced if a measles vaccine campaign is delivered but it will also depend on time delay of catch-up campaigns and speed at which a campaign can be organised. In December 2020 for instance, a SIA would reduce outbreak risk to zero in all scenarios with an upper bound risk of 15% while in December 2021, outbreak risk would reduce to zero with an upper bound risk of 25% after delivery of SIA.

The current disruption to vaccination services will cause further delays to vaccination, which is a challenge even in normal circumstances. We had previously reported consistently poor timeliness of MCV1 vaccination across 6 different birth-cohorts (2011–2016) in Kenya [18]. Here, a delay in age of MCV1 by 3 months resulted in a marginal increase in outbreak risk. For instance, assuming a 50% reduction in routine vaccination, a delay in vaccination would see the risk increase from 46% (30–59) to 53% (40–64) by the end of the year. This reiterates the importance of timeliness in administration of vaccines in children as even a slight delay may cause considerable immunity gaps.

Our results emphasise the importance of maintaining high RI coverage during this pandemic because the benefits of sustaining RI services far outweighs the risks of any excess COVID-19 deaths that may arise from vaccination clinic visits [5]. Due to the highly infectious nature of measles, massive outbreaks following disruptions to health care systems and reduced MCV1 coverage are typical. Following the West Africa Ebola outbreak in 2014–2015, Liberia, Sierra Leone, and Guinea reported more than a 25% reductions in MCV1 coverage [26, 27]. Reported cases also occurred in a lower age group compared to pre-Ebola period suggesting accumulation of susceptible children who missed their vaccine doses was a key contributor. Immunity gaps continued to be felt in these countries 2 years later even after successful implementation of SIAs.

Recently, measles outbreaks have been reported in five counties in Kenya [28] even with COVID-19 restrictions which suggests an adverse synergistic interaction between pre-existing gaps of susceptibility due to lower vaccination coverage in some counties (compared to national estimates) and a precipitous drop in RI coverage during this period. These outbreaks and our results are well aligned with recent Kenya measles risk assessment report by the Measles and Rubella Initiative, and recent WHO guidance on catch-up vaccination to close the immunity gaps caused by the COVID-19 pandemic.

As expected, majority of vaccine eligible children had protective antibody concentrations against measles while only one of 20 (0.05%) infants under 9 months old had protective levels. This suggests that there is an extended period of susceptibility in young infants probably a consequence of rapid decay of maternally acquired antibody. This will require further investigation in Kenya. However, this phenomenon has been previously reported in areas where maternal immunity is increasingly from immunisation rather than natural infection [29].

Our analysis was based on data from a rural area in the African region. Although these results are largely representative of rural areas in measles endemic settings, they may vary in an urban setting especially as measles susceptibility profiles have been shown to vary across urban and rural settings mainly due to heterogeneity in vaccination coverage and the different mixing patterns between and within age-groups.

A key strength of our study is the availability of recent serological data which provides an excellent means of directly estimating levels of population protection against infection and can also be used to guide post-COVID-19 SIAs. In addition, the availability of an age-mixing matrix from the same area allowed us to estimate overall immunity by taking into account the level of contact between different age-groups.

Our study has a few limitations. Population immunity was only available for children < 15 years, but we varied observed immunity estimates in adults from a previous study in our model which resulted in a slight shift in overall immunity. Our results showing SIAs conducted in under-fives will mitigate the risk of measles outbreak risk are based on the assumption that majority (96%) of the older age groups have measles immunity. Susceptibility gaps in this older age-groups will require SIAs for a wider age range (e.g. 9 months to 15 years) to close population immunity gaps and reduce the outbreak risk. The serological data estimates and the mixing matrix used in our study may not be fully representative of the country although we utilised national estimates of vaccination coverage, which was the main driver of predicted immunity. We did not explicitly model MCV2 delivery but assumed the overall effectiveness was an average of MCV1 and MCV2 efficacy weighted by the proportion of children who either receive MCV1 only or both doses. Finally, there is some uncertainty around the actual reduction in transmission due to variability in compliance with physical distancing measures in place. However, we accounted for uncertainty by varying both the reduction in transmission and the R0.

## Conclusions

Measles SIA originally scheduled for February 2020 in Kenya would have been well-timed as population immunity was below herd immunity threshold. Interruptions to RI since the start of COVID-19 pandemic restrictions in Kenya have now widened the measles immunity gap, but the associated risk of large measles outbreaks were partially mitigated by COVID-19 contact restrictions in place. As these measures have almost been fully lifted, we estimate that measles outbreak risks will dramatically increase, and an immediate SIA will be required to close measles immunity gaps.

## Supplementary Information

**Additional file 1.** Serological data used in the analysis.

**Additional file 2.** Age stratified population immunity profiles for age-adjusted immunity.

**Additional file 3.** Age-adjusted immunity simulations with proportion immune greater than herd immunity threshold.

**Additional file 4.** Outbreak probability for different scenarios of reduction in measles transmissibility.

**Additional file 5.** Impact of SIA on outbreak probability for different scenarios of reduction in measles transmissibility.

**Additional file 6.** Impact of delayed vaccination on outbreak probability.

**Additional file 7.** Monthly projected crude versus age-adjusted immunity profiles from September 2019 to December 2021.

**Additional file 8.** Crude immunity simulations with proportion immune greater than herd immunity threshold.

## Data Availability

All analyses were done in R [30] and are available on GitHub at: https://github.com/CarolineNM/ncov_measles_Kenya.
