# Supplementary material for: The importance of supplementary immunisation activities to prevent measles outbreaks during the COVID-19 pandemic in Kenya
Source: BMC Med. 2021 Feb 3;19:35. doi: 10.1186/s12916-021-01906-9 (PMC7854026; doi:10.1186/s12916-021-01906-9)
Supplement: Supplementary file 1 — Additional file 1. Serological data used in the analysis. [file 12916_2021_1906_MOESM1_ESM.docx]

**Table S1. Serological data used in the analysis. The table shows counts of all tested individuals and positive individuals in the different age-categories**

| Age-categories | All samples | Positive samples |
| --- | --- | --- |
| <9m | 20 | 1 |
| 9m-<1yr | 18 | 9 |
| 1yr | 48 | 45 |
| 2yrs | 47 | 42 |
| 3yrs | 53 | 49 |
| 4-8yrs | 237 | 228 |
| 9-14yrs | 74 | 71 |
| Total | 497 | 445 |
