# Supplementary material for: The importance of supplementary immunisation activities to prevent measles outbreaks during the COVID-19 pandemic in Kenya
Source: BMC Med. 2021 Feb 3;19:35. doi: 10.1186/s12916-021-01906-9 (PMC7854026; doi:10.1186/s12916-021-01906-9)
Supplement: Supplementary file 2 — Additional file 2. Age stratified population immunity profiles for age-adjusted immunity. [file 12916_2021_1906_MOESM2_ESM.docx]

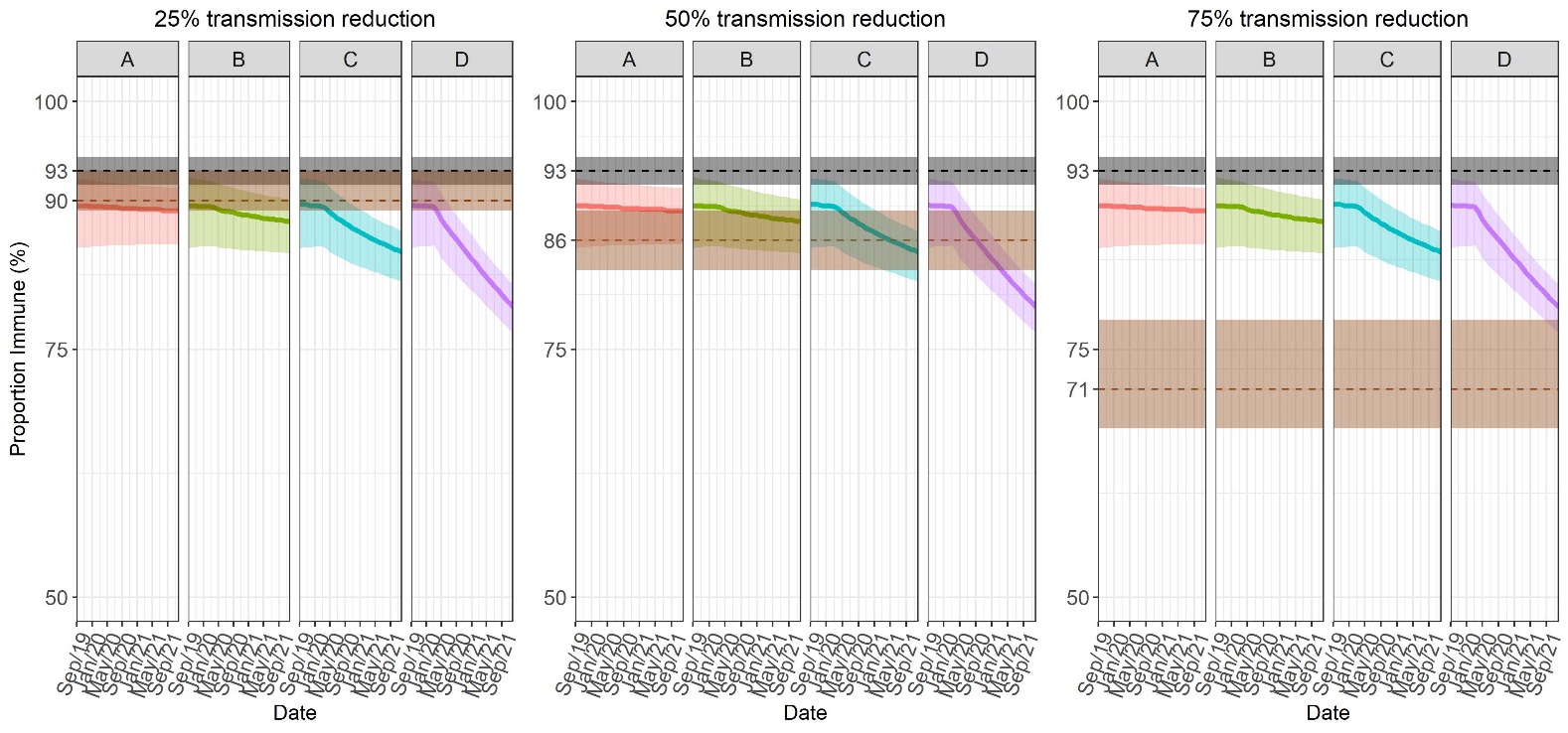


Figure S1. Age stratified population immunity profiles. The three HITs are based on the three assumptions of reduction in transmission of measles during the pandemic
