# Supplementary material for: The importance of supplementary immunisation activities to prevent measles outbreaks during the COVID-19 pandemic in Kenya
Source: BMC Med. 2021 Feb 3;19:35. doi: 10.1186/s12916-021-01906-9 (PMC7854026; doi:10.1186/s12916-021-01906-9)
Supplement: Supplementary file 4 — Additional file 4. Outbreak probability for different scenarios of reduction in measles transmissibility. [file 12916_2021_1906_MOESM4_ESM.docx]

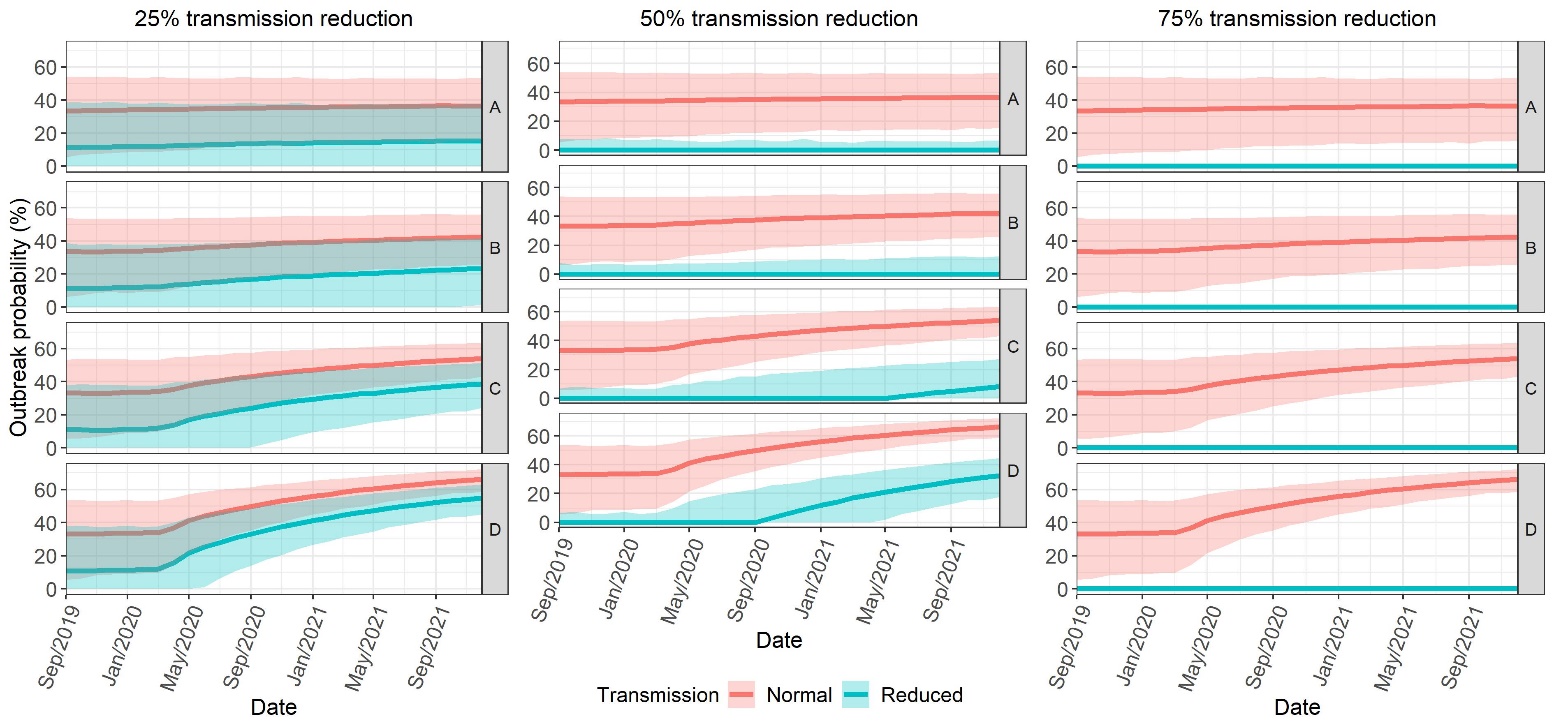


Figure S1. Probability of a large outbreak sparked by a single infectious individual assuming different levels of reduction in measles transmission during the pandemic
