# Supplementary material for: The importance of supplementary immunisation activities to prevent measles outbreaks during the COVID-19 pandemic in Kenya
Source: BMC Med. 2021 Feb 3;19:35. doi: 10.1186/s12916-021-01906-9 (PMC7854026; doi:10.1186/s12916-021-01906-9)
Supplement: Supplementary file 5 — Additional file 5. Impact of SIA on outbreak probability for different scenarios of reduction in measles transmissibility. [file 12916_2021_1906_MOESM5_ESM.docx]

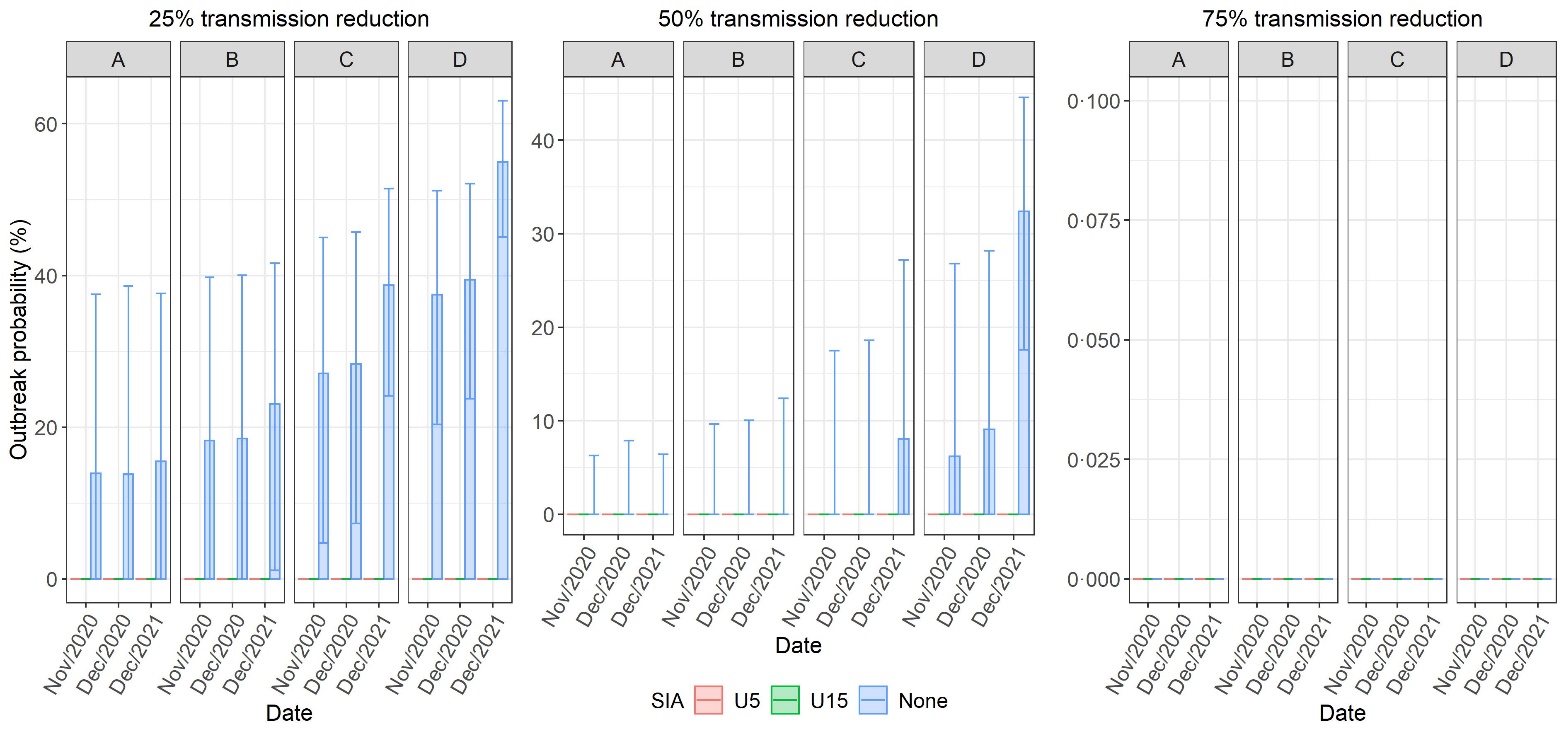


Figure S1. Probability of a single infectious person seeding a large outbreak before (none) and after implementing a SIA in children 9 months to 5 years old (U5) and in 9 months to 15 years old (U15) at different timepoints during lockdown (25%, 50% and 75% reduction in measles transmission)
