# Supplementary material for: The importance of supplementary immunisation activities to prevent measles outbreaks during the COVID-19 pandemic in Kenya
Source: BMC Med. 2021 Feb 3;19:35. doi: 10.1186/s12916-021-01906-9 (PMC7854026; doi:10.1186/s12916-021-01906-9)
Supplement: Supplementary file 6 — Additional file 6. Impact of delayed vaccination on outbreak probability. [file 12916_2021_1906_MOESM6_ESM.docx]

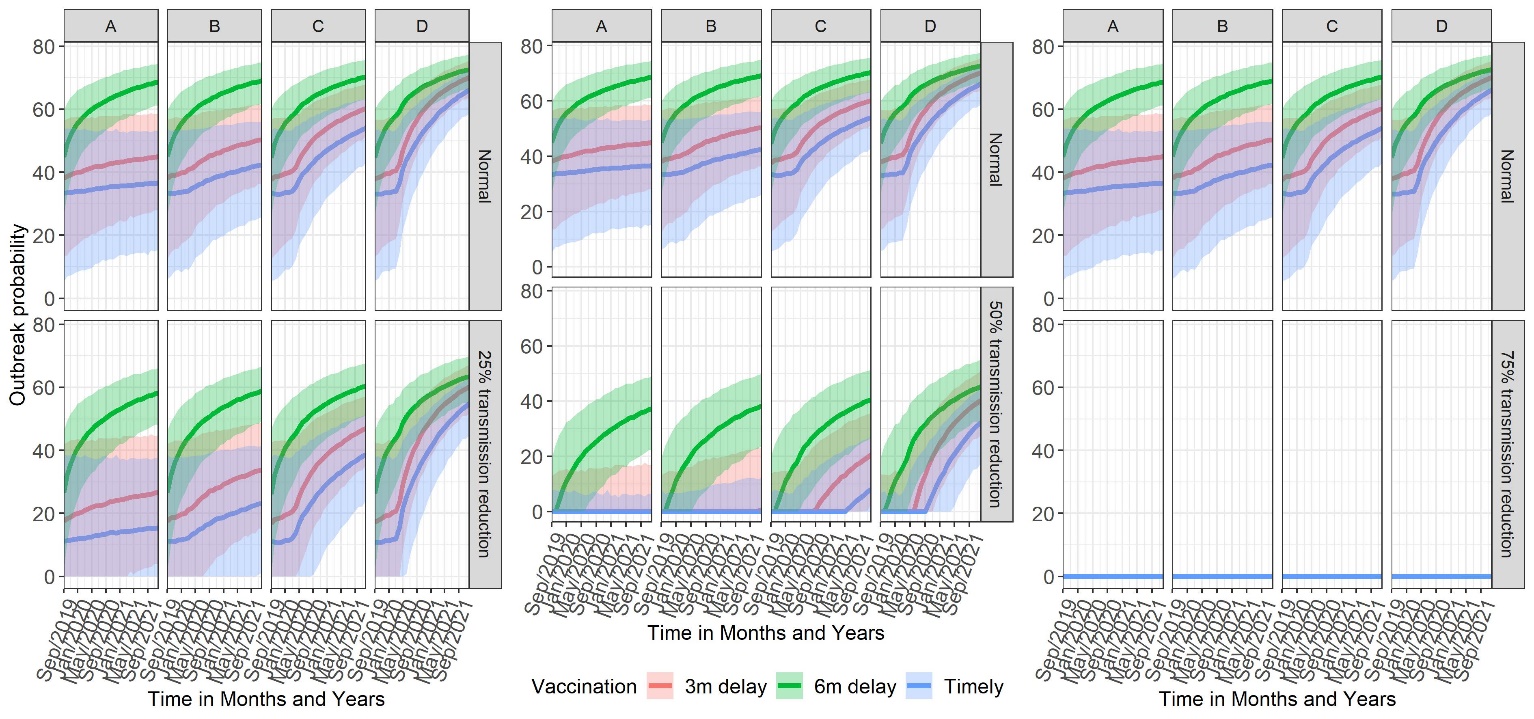


Figure S1. Impact of delayed vaccination on outbreak probability after lifting of contact reducing measures (Normal transmission) and assuming different levels of reduction in measles transmission during the pandemic.
