# Supplementary material for: The importance of supplementary immunisation activities to prevent measles outbreaks during the COVID-19 pandemic in Kenya
Source: BMC Med. 2021 Feb 3;19:35. doi: 10.1186/s12916-021-01906-9 (PMC7854026; doi:10.1186/s12916-021-01906-9)
Supplement: Supplementary file 7 — Additional file 7. Monthly projected crude versus age-adjusted immunity profiles from September 2019 to December 2021. [file 12916_2021_1906_MOESM7_ESM.docx]

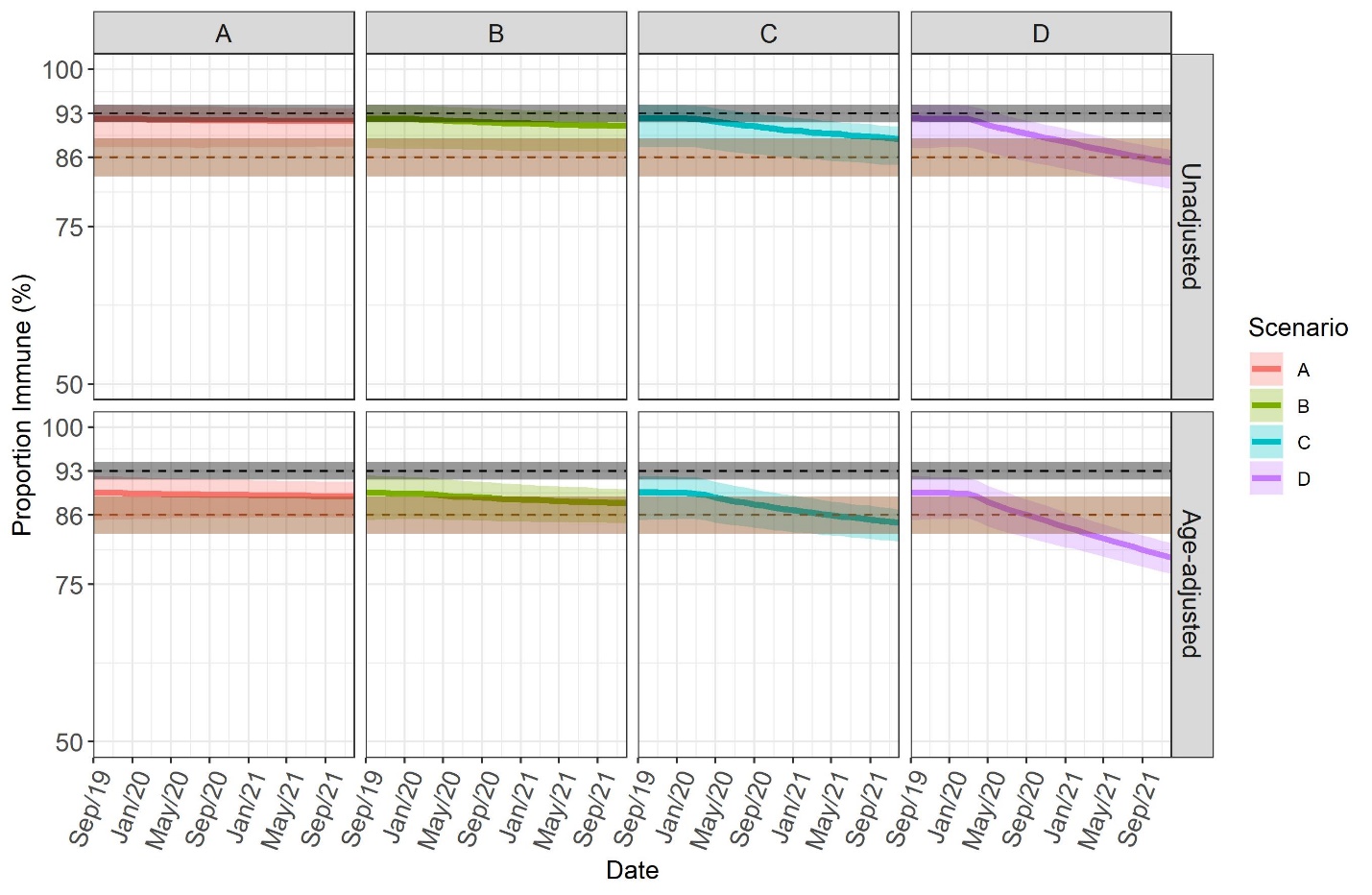


**Figure S1. Monthly projected unadjusted and contact adjusted immunity profiles from September 2019 to December 2021.** The changes in coverage took effect in April 2020∙ The black line shows the herd immunity threshold for measles before the COVID-19 pandemic 0∙93 (0∙92 to 0∙94) and the brown line shows the herd immunity threshold during COVID-19 pandemic of 50%, 0∙86 [0∙83-0∙89], assuming the lockdown measures are still in effect
