# Supplementary material for: The importance of supplementary immunisation activities to prevent measles outbreaks during the COVID-19 pandemic in Kenya
Source: BMC Med. 2021 Feb 3;19:35. doi: 10.1186/s12916-021-01906-9 (PMC7854026; doi:10.1186/s12916-021-01906-9)
Supplement: Supplementary file 8 — Additional file 8. Crude immunity simulations with proportion immune greater than herd immunity threshold. [file 12916_2021_1906_MOESM8_ESM.docx]

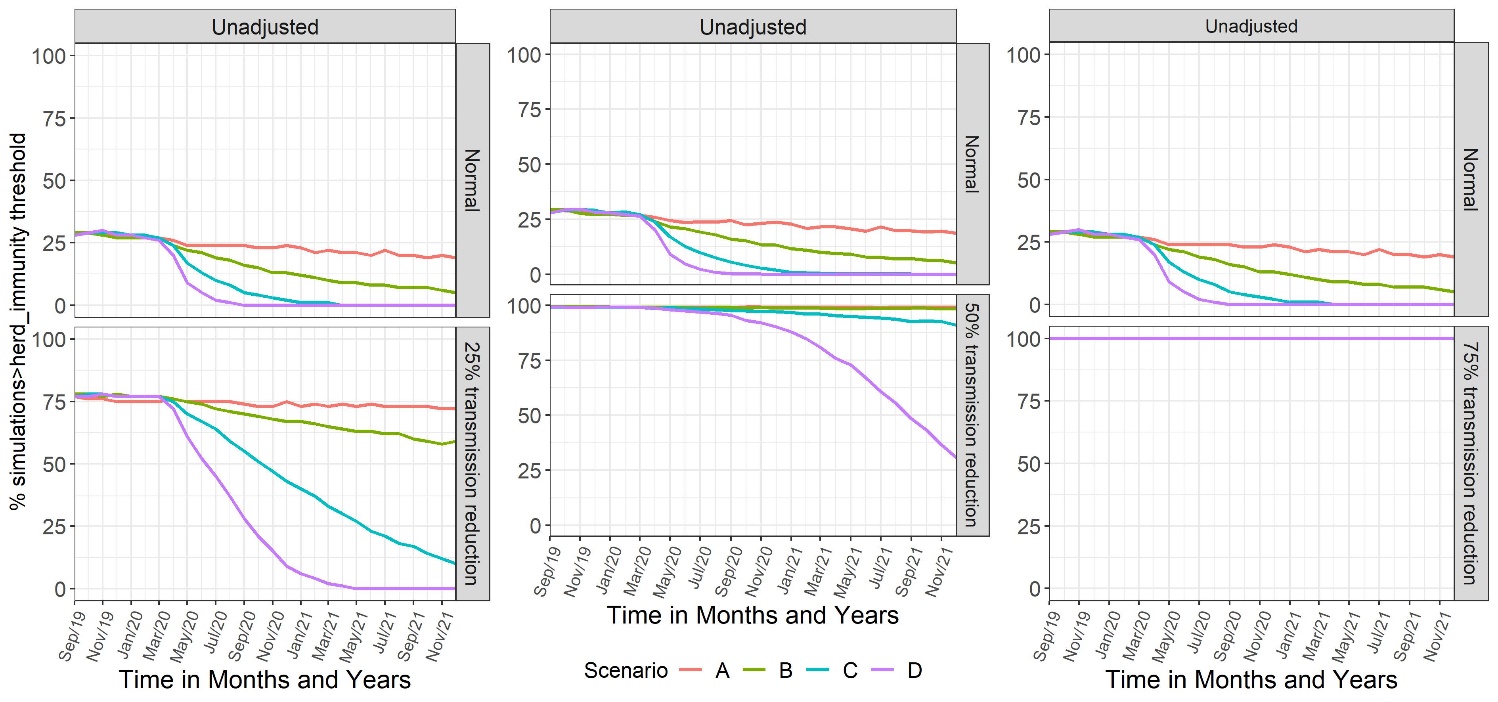


**Figure S1. Percentage of simulations with proportion immune > herd immunity threshold for crude population immunity.**
